# The Characterization of the Endoglucanase Cel12A from *Gloeophyllum trabeum* Reveals an Enzyme Highly Active on β-Glucan

**DOI:** 10.1371/journal.pone.0108393

**Published:** 2014-09-24

**Authors:** Lis Schwartz Miotto, Camila Alves de Rezende, Amanda Bernardes, Viviane Isabel Serpa, Adrian Tsang, Igor Polikarpov

**Affiliations:** 1 Grupo de Biotecnologia Molecular, Instituto de Física de São Carlos, Universidade de São Paulo, São Carlos, SP, Brazil; 2 Instituto de Química, Universidade Estadual de Campinas, Campinas, SP, Brazil; 3 Centre for Structural and Functional Genomics, Concordia University, Montreal, QC, Canada; Universidad de Granada, Spain

## Abstract

The basidiomycete fungus *Gloeophyllum trabeum* causes a typical brown rot and is known to use reactive oxygen species in the degradation of cellulose. The extracellular Cel12A is one of the few endo-1,4-β-glucanase produced by *G. trabeum*. Here we cloned *cel12A* and heterologously expressed it in *Aspergillus niger*. The identity of the resulting recombinant protein was confirmed by mass spectrometry. We used the purified GtCel12A to determine its substrate specificity and basic biochemical properties. The *G. trabeum* Cel12A showed highest activity on β-glucan, followed by lichenan, carboxymethylcellulose, phosphoric acid swollen cellulose, microcrystalline cellulose, and filter paper. The optimal pH and temperature for enzymatic activity were, respectively, 4.5 and 50°C on β-glucan. Under these conditions specific activity was 239.2±9.1 U mg^−1^ and the half-life of the enzyme was 84.6±3.5 hours. Thermofluor studies revealed that the enzyme was most thermal stable at pH 3. Using β-glucan as a substrate, the K_m_ was 3.2±0.5 mg mL^−1^ and the V_max_ was 0.41±0.02 µmol min^−1^. Analysis of the effects of GtCel12A on oat spelt and filter paper by scanning electron microscopy revealed the morphological changes taking place during the process.

## Introduction

Cellulases are hydrolytic enzymes that can be classified into three main categories according to their activities: endoglucanases, or endo-1-4-ß-glucanases, which cleave the cellulose internally and expose new free ends in the polymer; exoglucanases, or cellobiohydrolases, which act processively from the free ends to release soluble cellobiose molecules; and ß-glucosidases which hydrolyze cellobiose into glucose [1], [2]. These enzymes are classified into glycoside hydrolase (GH) families, see http://www.cazy.org/Glycoside-Hydrolases.html [3] with different structural and functional features.

In aerobic fungi, endoglucanases and cellobiohydrolases can be found in the following GH families: GH5, GH6, GH7, GH9, GH12, GH45 and GH74. A search of the database containing characterized lignocellulolytic enzymes of fungal origin, https://mycoclap.fungalgenomics.ca [4], revealed that over 60% of the endoglucanases and cellobiohydrolases belonging to the families GH5, GH6, GH7, GH45 and GH74 are modular multi-domain proteins. They possess a carbohydrate-binding module (CBM) that is separated from the catalytic domain by an interdomain linker. In contrast, all 34 enzymes from the GH12 family have a catalytic domain only [5]. Members of GH12 enzymes are involved in the hydrolysis of β-1,4 glycosidic linkages of cellulose via double displacement reaction mechanism that results in the retention of the anomeric configuration of the product [6], [7]. Endoglucanase, xyloglucan hydrolase, and β-1,3-1,4-glucanase are activities included in the GH12 family [3].

*Gloeophyllum trabeum* is a brown-rot basidiomycete fungus. Brown-rot fungi consume the cellulose in wood and leave behind the brown rotted wood that is composed mostly of lignin. Brown-rot fungi use both extracellular reactive oxygen species and hydrolytic enzymes to degrade cellulose [8]. The genome sequence of *G. trabeum* has been published [9]. Like most brown-rot fungi, *G. trabeum* does not harbor genes for GH6 and GH7 proteins and therefore do not have cellobiohydrolases. Genes predicted to encode endoglucanases in the *G. trabeum* genome are two GH5 endoglucanases, two GH12 proteins and one each of GH45 and GH74 proteins. Two of the genes, *cel5A* and *cel12A*, have been characterized previously [10]. The GtCel5A enzyme was shown to be able to hydrolyze crystalline cellulose, while GtCel12A is active on carboxymethyl cellulose (CMC), which has an amorphous structure. The paucity of cellulase genes and the lack of cellobiohydrolase genes in the genome make the few cellulases of *G. trabeum*, attractive enzymes for detailed characterization as they may possess functionality that have not been observed previously. The relatively small size, <30 kDa, and the lack of CBM of GH12 enzymes suggest that they may be able to penetrate the low-porosity wood to depolymerize cellulose. Fungal GH12 enzymes in general have not been thoroughly characterized. These considerations prompted us to investigate further the role of GtCel12A in cellulose degradation. In this study we report the expression of *cel12A* of *G. trabeum* in *Aspergillus niger*. We describe the purification and biochemical characterization of the recombinant enzyme. We also report the scanning electron microscopy analysis of substrates following hydrolysis by GtCel12A.

## Materials and Methods

### 1. Gene cloning and recombinant expression

Complementary DNA was prepared by reverse transcription of total RNA isolated from *Gloeophyllum trabeum* strain ATCC 11539 grown in a medium containing barley and alfalfa 1% (w/v) for 32 hours at 34°C. The gene sequence was selected from GenBank (accession number HQ163778). The recombinant expression was carried out through the gene amplification from the cDNA by PCR using three primers as follows: 5′-TACTTCCAATCCAATCCATTTGACGATATGTTCCGCTTCATCTCTGCTTTGCCCTTTG-3′ (forward), 5′-ATGATGATGATGATGATGGGATCCACGCGGAACCAGCCCGCTCAAGCTGAC-3′ (reverse 1) and 5′-TTATCCACTTCCAATCCATTTGTTAATGATGATGATGATGATGGGATCCACGCGGAACCA-3′ (reverse 2).

Both insert and vector were amplified with specific primers designed to enable the cloning using the ligation-independent cloning method as previously described [11]. The LIC ANIp7G, a vector constructed from ANIp7 [12] was treated with T4 DNA polymerase in the presence of only dCTP, whereas dGTP was used for the insert. Annealing of the insert and ANIp7G molecules, both carrying complementary ends, was carried out at room temp for 1 hour.

Recombinant DNA was transformed into *Aspergillus niger* py11 (*cspA^−^ pyrG^−^* Δ*Gla::hiG*), derived from strain N593 (*cspA^−^ pyrG^−^*), using the protoplast transformation method as previously described [13]. Transformants were selected on minimal medium (MM) [14] without uracil and uridine. The ANIp7G vector without the inserted DNA was included as control.

### 2. Protein production and purification

Transformants were cultured in MM J media, which is similar to MM except that it includes 4% (w/v) maltose as the sole carbon source and the amounts of nitrogen source, salts and trace elements were increased by 4-fold. Six colonies from the transformation plates were randomly selected and the conidia were transferred to 24 wells plates containing 1 mL of MM J and grown for 6 days at 30°C without agitation. After the incubation period, the supernatant of the cultures was harvested and analyzed by SDS-PAGE to confirm positive protein production.

For protein purification, 2-L Erlenmeyers flasks containing 500 mL of MM J media were inoculated with positive transformants at 2×10^6^ conidia mL^−1^ and the culture was grown for 6 days at 30°C without agitation. Culture supernatant was filtered through Miracloth (Merck KGaA, Darmstadt, Germany) and then precipitated with 80% of ammonium sulfate at 10°C. The pellet was resuspended in 50 mM sodium citrate buffer (pH 3.0) and washed several times with the same buffer. The resulting supernatant was further applied to a desalting column, Sephadex G25 (GE Healthcare, Little Chalfont, UK). Then, the desalinated protein sample was applied to an ion-exchange SP Sepharose (GE Healthcare, Little Chalfont, UK) column equilibrated with the same buffer as a first purification step. Proteins were eluted with a linear gradient of 0–1 M of sodium chloride in the same buffer at a flow rate of 1 mL min^−1^. The samples were analyzed by SDS-PAGE and the fractions containing the protein of interest were combined and concentrated using a 10 kDa cut-off Vivaspin (GE Healthcare, Little Chalfont, UK) concentrator. In a second purification step, the concentrated volume was applied to a Superdex 75 10/30 column (GE Healthcare, Little Chalfont, UK), equilibrated with the same buffer added of 0.2 M of sodium chloride. Fractions (1 mL) were collected at a flow rate of 1 mL min^−1^ and were assayed for activity on β-glucan and CMC. All chromatographic steps were performed using AKTA chromatography system (GE Healthcare, Little Chalfont, UK) at 4°C and fractions were stored at the same temperature until further use.

To confirm the experimental molecular weight of GtCel12A, a calibration curve was assembled using a molecular exclusion column, Superdex 75 (GE Healthcare, Little Chalfont, UK). Standard molecules were applied and the respective elution volumes were calculated. The standard molecules used were: blue dextran (2000 kDa), conalbumin (75 kDa), egg albumin (44 kDa), carbonic anhydrase (29 kDa), ribonuclease A (13.7 kDa) and aprotinin (6.5 kDa), all purchased from GE Healthcare.

### 3. Mass Spectrometric Identification

After analysis of the protein production by SDS-PAGE, the Coomassie Blue-stained band corresponding to GtCel12A was submitted to in-gel digestion with 0.5 µg of modified trypsin for 16 hours (Promega, Madison, USA). The tryptic peptides were extracted with a solution containing formic acid (5%) and acetonitrile (50%) and further submitted to LC/MS analysis using MicroTOF-QII (Bruker Daltonics, Billerica, USA) mass spectrometer. The mass spectra of peptides generated by mono-isotropic peptide masses obtained were interpreted using the Mascot search engine (http://www.matrixscience.com/serv​er.html; Matrix Science, Boston, USA). The peptide sequences were compared with the non-redundant databases of filamentous fungi proteins generated from data compiled by the Joint Genome Institute.

### 4. Substrate specificity

Substrate specificity was examined in assays using a range of 21 substrates. Substrates were prepared as 1% stock solutions in MilliQ water, except for the amorphous cellulose which was suspended at 5.7 mg mL^−1^ in sodium acetate buffer, pH 4.6. For filter paper, we used a 0.7 cm disc of Whatman No. 1. The tested substrates were: arabinoxylan from wheat flour, arabinoxylan from rye flour, β-glucan from barley, lichenan from icelandic moss, xylan from oat spelt, linear 1,5-α-L-arabinan from sugar beet, arabinogalactan from larch wood, 1,4-β-D-mannan, laminarin, debranched arabinan from sugar beet, galactomannan, xyloglucan from tamarind (all purchased from Megazyme, Wicklow, Ireland), filter paper (Whatman, Little Chalfont, UK), hydroxyethylcellulose, Avicel PH-101 (microcrystalline cellulose) (Fluka, St Louis, USA), 4-Nitrophenyl β-D-cellobioside (4NPC), 4-Nitrophenyl β-D-glucopyranoside (4NPG), CMC, xylan from beechwood, Sigmacell 20 (microcrystalline cellulose) (Sigma, St Louis, USA) and phosphoric acid swollen cellulose (PASC) [15].

Each assay was performed in triplicate in a final volume of 100 µL containing: 50 µL of each substrate at 1% (w/v), 1.5 µg of enzyme in 5 µL, and 45 µL of 50 mM sodium citrate buffer, pH 4.5. For control, the volume of enzyme was replaced with 5 µL of the same buffer. The reaction mixture was incubated at 5°C for 30 minutes and 100 µL of dinitrosalicilic acid (DNS) [16] was added followed by a 5-minute incubation at 95°C. Absorbance was measured at 540 nm.

For reactions with 4NPG and 4NPC, after the incubation time of 30 minutes, 100 µL of 1M Na_2_CO_3_ was added, followed by absorbance measurements at 400 nm.

The amount of reducing sugars was determined using the DNS method [17]. One unit of the enzyme was defined as 1 µmol of glucose equivalent released per minute under the assay conditions.

### 5. Determination of the optimal conditions for activity

Temperature and pH profiles of the enzymatic activity were performed using β-glucan from barley at 1% (w/v) as substrate. The final reaction volume was 100 µL, which contained 0.15 µg of enzyme in 5 µL, 45 µL of each buffer of pH values ranging from 1 to 10 at 50 mM, and 50 µL of the substrate diluted in water. In the reaction control, the volume of enzyme was replaced with 5 µL of the same buffer. Initially, a screening of the optimal conditions was performed using ABP (acetate/borate/phosphate) buffer, with pH values ranging from 2 to 10. Then, different buffers were used to confirm the preliminary results observed (data not shown). All buffers were prepared at 50 mM. For pH 1, we used hydrochloric acid at 50 mM and for pH 2, hydrochloric acid equilibrated with potassium chloride. Sodium citrate buffer was used for pH 3, 3.5, 4.0, 4.5, 5.0 and 6.0, sodium phosphate for pH 7, Tris/HCl for 8 and 9, and sodium carbonate/bicarbonate for pH 10. Each assay was performed in triplicate. The experiment was performed in 96 wells PCR plates (BioRad, Hercules, USA) using a thermocycler (BioRad, Hercules, USA) with a temperature gradient function, and temperature ranging from 30 to 80°C for all pH values, totalizing 768 samples. After determining the optimal conditions of pH and temperature, separately, we also investigated the influence of enzyme concentration on the enzymatic assay (data not shown).

Residual enzymatic activity was determined under the optimal conditions found in the previous experiment and was performed by incubating the enzyme diluted in 50 mM of sodium citrate buffer, pH 4.5 at 50°C for 168 hours. Aliquots of 5 µL were removed and assayed for activity on β-glucan from barley at 1% over a time course of 168 h.

### 6. Thermal stability analysis using Thermofluor

To investigate the effect of pH on thermal stability for GtCel12A, 10 µM of protein in different buffer solutions were mixed with 1 µL of Sypro Orange (Invitrogen, Carlsbad, USA), used as the reporter dye, with a final concentration of 1/2000. All buffers used in the analysis were prepared at 50 mM. All measurements were performed in triplicate in a final volume of 20 µL. In the reaction control, the volume of enzyme was replaced with 19 µL of the same buffer. Mixtures were added to a 96-well thin wall PCR plate (Bio-Rad, Hercules, USA) and sealed with Optical-quality sealing tape (Bio-Rad, Hercules, USA) and incubated in an iCycler iQ Real-Time PCR Detection System (Bio-Rad, Hercules, USA). The temperature range tested was from 25 to 90°C, with stepwise increments of 1°C per minute and 10 s hold step for every point, followed by the fluorescence reading with excitation/emission wavelengths at 490/530 nm. Melting curve analysis and the melting temperature (T_m_) determination were carried out using GraphPad Prism software (version 5.0) (GraphPad Software, La Jolla, USA).

### 7. Enzyme kinetics

The kinetic parameters were determined using β-glucan from barley as a substrate. The reaction volume was 100 µL, which contained 50 µL of each substrate to yield final substrate concentrations of 0.6, 1.25, 2.50, 5, 10 and 15 (mg mL^−1^), 45 µL of sodium citrate buffer, pH 4.5 and 5 µL of enzyme. The final enzyme concentration used in the experiment was 30 nM.

The 100 µL reaction mixtures were incubated in a 96 well micro plate at 50°C. Triplicates were removed after 1, 2, 5, 10, 12 and 15 min and were immediately assayed for reducing sugar end determination by DNS. The K_m_ and V_max_ parameters for each substrate were determined by Michaelis-Menten fitting using the Origin v 8.6 software programme (OriginLab, Northampton, USA).

### 8. Scanning electron microscopy (SEM)

Samples of oat spelts, a β-glucan rich substrate, and filter paper were hydrolyzed by GtCel12A for 12 hours (oat spelt and filter paper) and 24 hours (only filter paper) at 50°C and analyzed by SEM. The assays were performed in triplicate in a final volume of 100 µL, which contained 5 µg of enzyme in 5 µL, 95 µL of 50 mM sodium citrate buffer, pH 4.5 and 6 mg of oat spelts or a 0.7 cm disc of Whatman No. 1 filter paper. In the reaction control, the volume of enzyme was replaced with 5 µL of the same buffer. Following hydrolysis the substrates were washed with 2 mL of 50 mM sodium citrate buffer, pH 4.5 and dried at 37°C for 12 hours. After drying, the substrates were coated with gold in a SCD 050 sputter coater (Oerlikon-Balzers, Balzers, Lichtenstein). Sample imaging was carried out using a high resolution environmental scanning electron microscope, equipped with a field emission gun (FESEM) Quanta 650 (FEI, Hillsboro,USA). Both the coater and the microscope were available at the National Laboratory of Nanotechnology (LNNano) in Campinas-SP, Brazil. Images were obtained under vacuum, using a 5 kV accelerating voltage and a secondary electron detector. At least 20 images per sample were obtained on different areas of the samples to ensure the reproducibility of the results.

## Results

### 1. Purified recombinant GtCel12A displays high specificity on β-glucan

**Figure 1:**
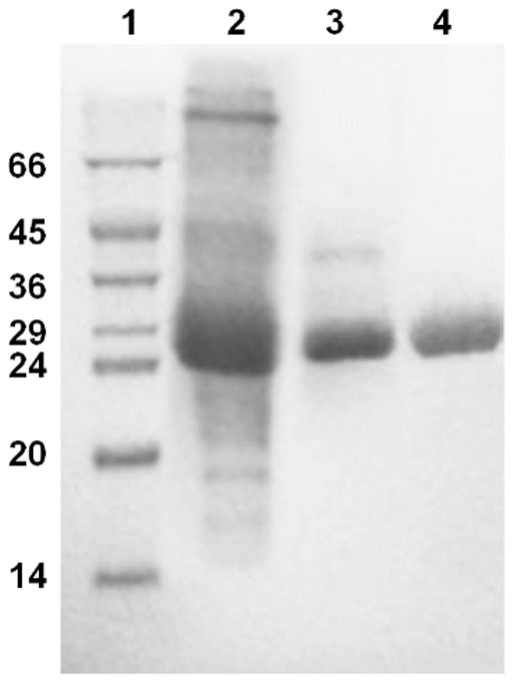
GtCel12A purification. 12% SDS-PAGE of proteins from the two purification steps. Lane 1, molecular weight markers; lane 2, culture broth; lane 3, proteins following SP Sepharose chromatography; and lane 4, protein after Superdex 75 column chromatography. The molecular weight standards are indicated in kDa.

Supernatant from the culture of one of the positive transformants was used to purify the recombinant Cel12A by SP Sepharose and Superdex 75 (GE Healthcare, Little Chalfont, UK) column chromatography. From the calibration performed on the molecular exclusion column, the experimental molecular weight of the protein was estimated to be close to 26.2±0.1 kDa, very close to the mass calculated from the amino acid residues of 26.1 kDa. This suggests that Cel12A is a monomeric enzyme with few or no post-translational modifications. Figure 1 shows that the enzyme following the molecular exclusion step is highly pure as determined by SDS-PAGE analysis.

**Figure 2:**
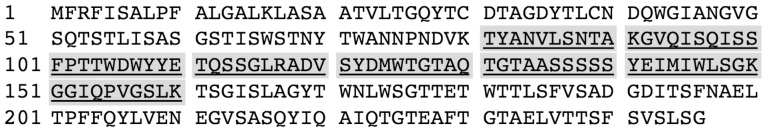
Mass spectrometric analysis of purified GtCel12A. Primary sequence of GtCel12A is shown with the peptides identified by LC-MS evidenced in gray.

The identity of the purified GtCel12A was confirmed by LC-MS analysis. Figure 2 shows that the tryptic peptides of the purified recombinant match to 32% of the *G. trabeum* Cel12A sequence.

**Figure 3:**
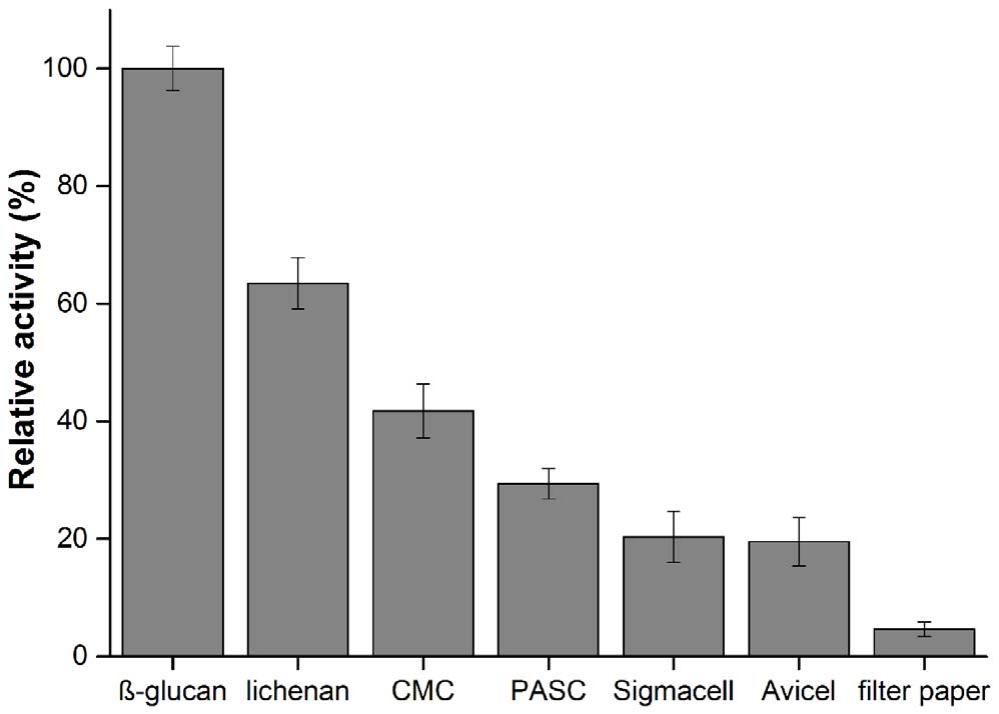
Substrate specificity for GtCel12A. Observed enzymatic activity was obtained on the following substrates: β-glucan, lichenan, CMC, PASC, Sigmacell, Avicel and filter paper.

**Figure 4:**
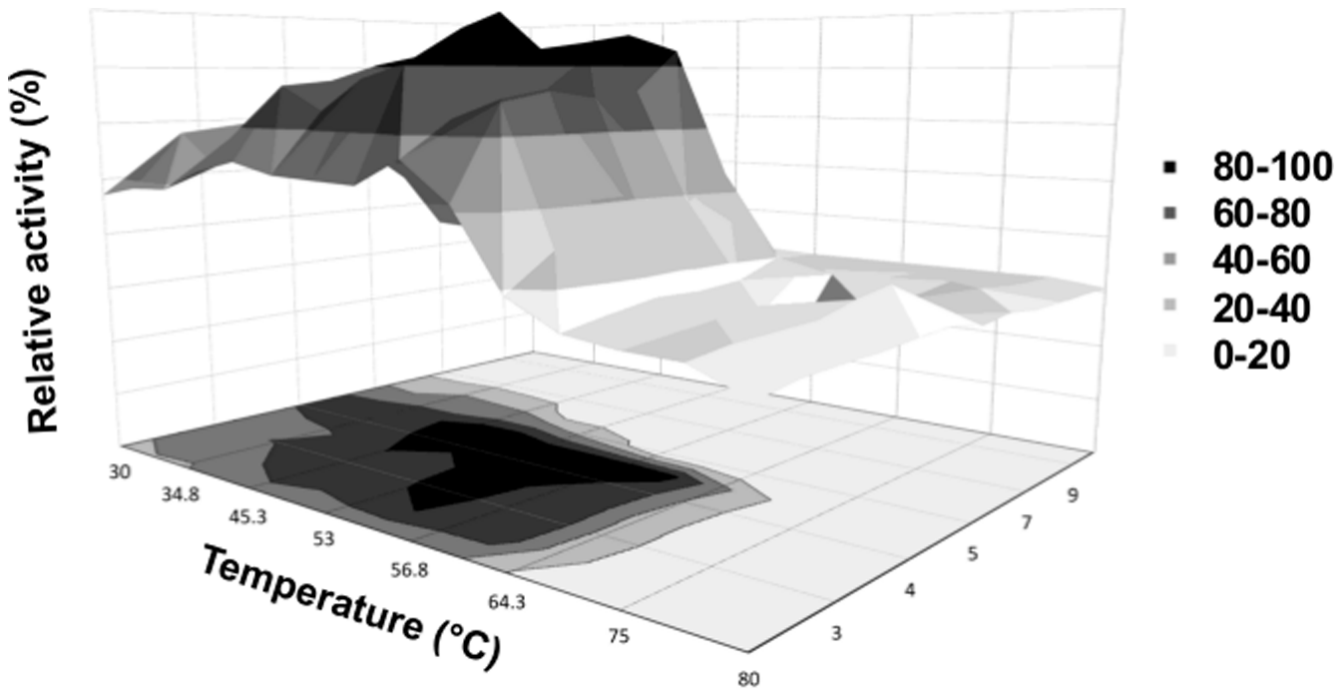
Optimal conditions (pH and temperature) for activity, represented in terms of relative activity. The temperature range is from 30 to 80°C, and pH from 1 to 10. The optimal conditions were pH 4.5 and 50°C.

**Figure 5:**
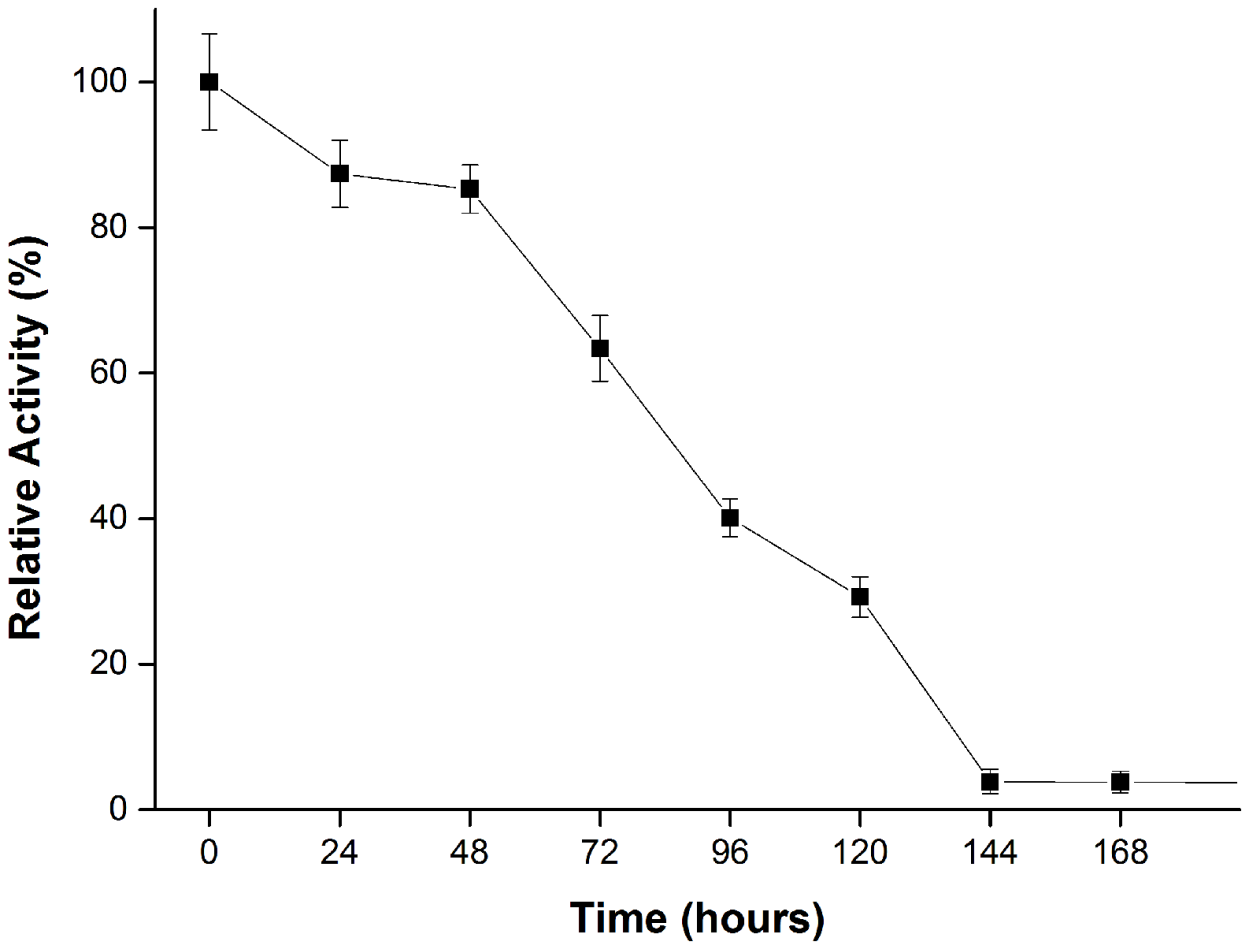
Residual enzymatic activity for GtCel12A. The assay was performed at pH 4.5 and temperature of 50°C, in sodium citrate buffer, over a time course of 168 hours.

The column-purified GtCel12A was assayed for activity with 21 different substrates. GtCel12A displayed the highest activity on β-glucan, and followed in descendent order by activity on lichenan, CMC, PASC, Avicel, Sigmacell and filter paper (figure 3). The optimal pH and temperature for the activity on β-glucan were 4.5 and 50°C, respectively (Figure 4). Thermal stability assay for GtCel12A reveals a half-life of 84.6±3.5 hours at 50°C and pH 4.5 and suggests that *Gloeophyllum trabeum* Cel12A is quite stable enzyme under the conditions of our study, since it retained about 30% of its activity even after 120 hours of incubation at 50°C (Figure 5). Under optimal pH, temperature and enzyme concentration, the highest specific activity of this enzyme was observed on β-glucan, which was 239.2±9.1 U mg^−1^. GtCel12A has K_m_ and V_max_ for β-glucan of 3.2±0.5 mg mL^−1^ and 0.40±0.02 µmol min^−1^, respectively.

### 2. GtCel12A is most thermal stable at pH lower than its optimal pH for activity

**Figure 6:**
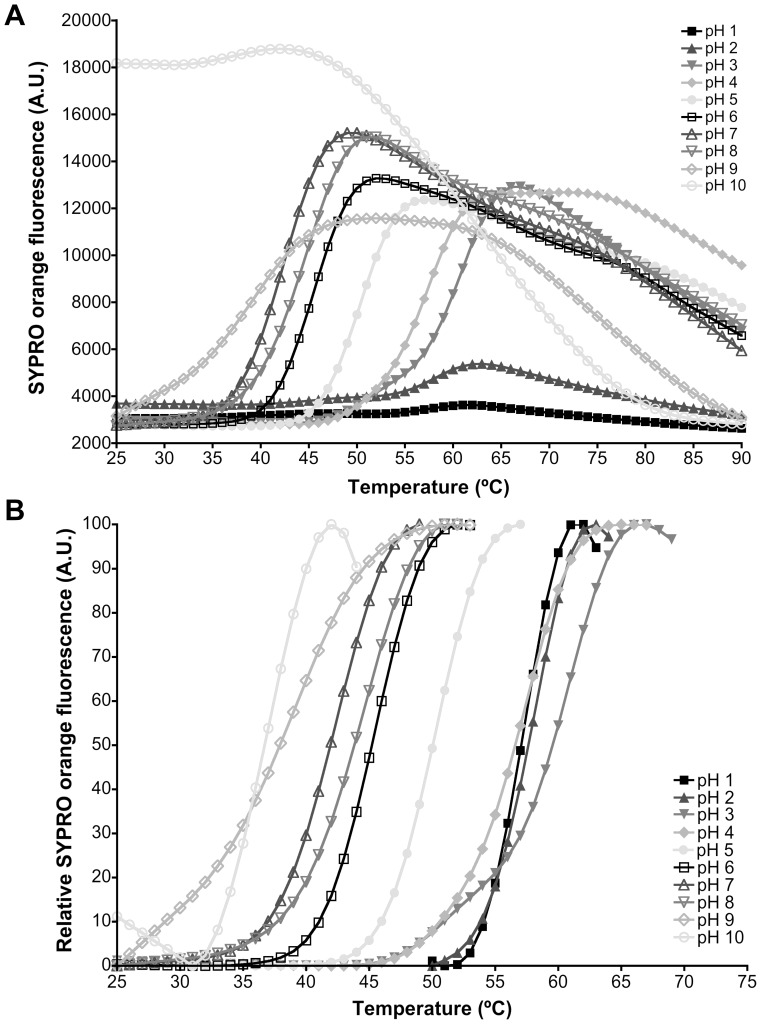
Thermofluor curves for GtCel12A. A. Plots of fluorescence intensity as a function of temperature and B. normalized data to visualize the thermal shift between different pH conditions.

**Figure 7:**
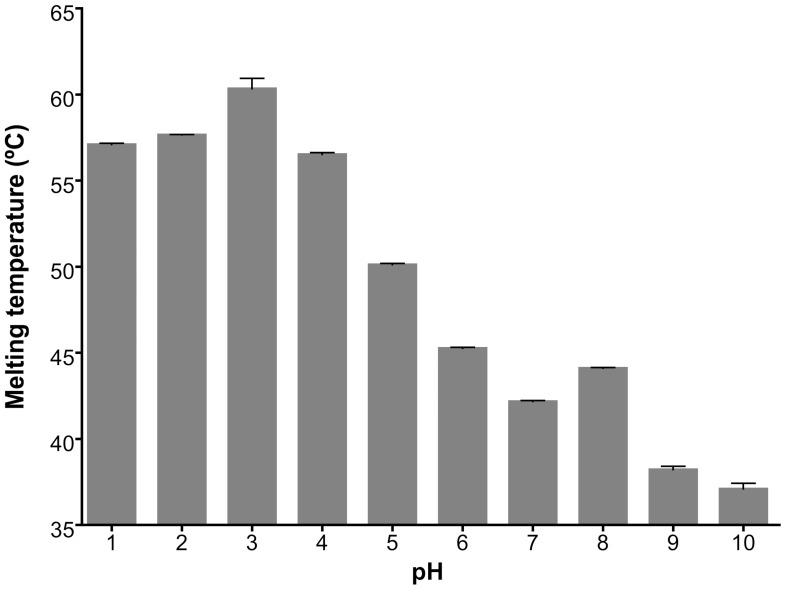
Variation of the melting temperature (T_m_) determined using the Boltzmann model for all tested pHs (1 to 10). The highest T_m_ value was observed for pH 3 (60. 4±0.6)°C.

The study of thermal stability using Thermofluor was performed in order to find the pH range where the protein would be stable in solution, and thus comparing with the optimal enzymatic activity condition. Although the optimum pH for enzymatic activity is 4.5, Thermofluor experiment revealed an optimum pH of 3.0 for enzyme stability (figure 6), since the highest T_m_ was observed for this pH value (figure 7), thereby confirming the acidophilic behavior of the enzyme. This might indicate that the enzyme requires some flexibility to explore a range of conformations required for its optimal activity. At pH 6.0 and higher values, the melting temperature decreased dramatically, suggesting a loss of protein stability. At pH 10, the protein, even at low temperatures, displayed characteristics of unfolded protein (figure 6).

### 3. Analysis of the effects of GtCel12A on oat spelt and crystalline cellulose by SEM

**Figure 8:**
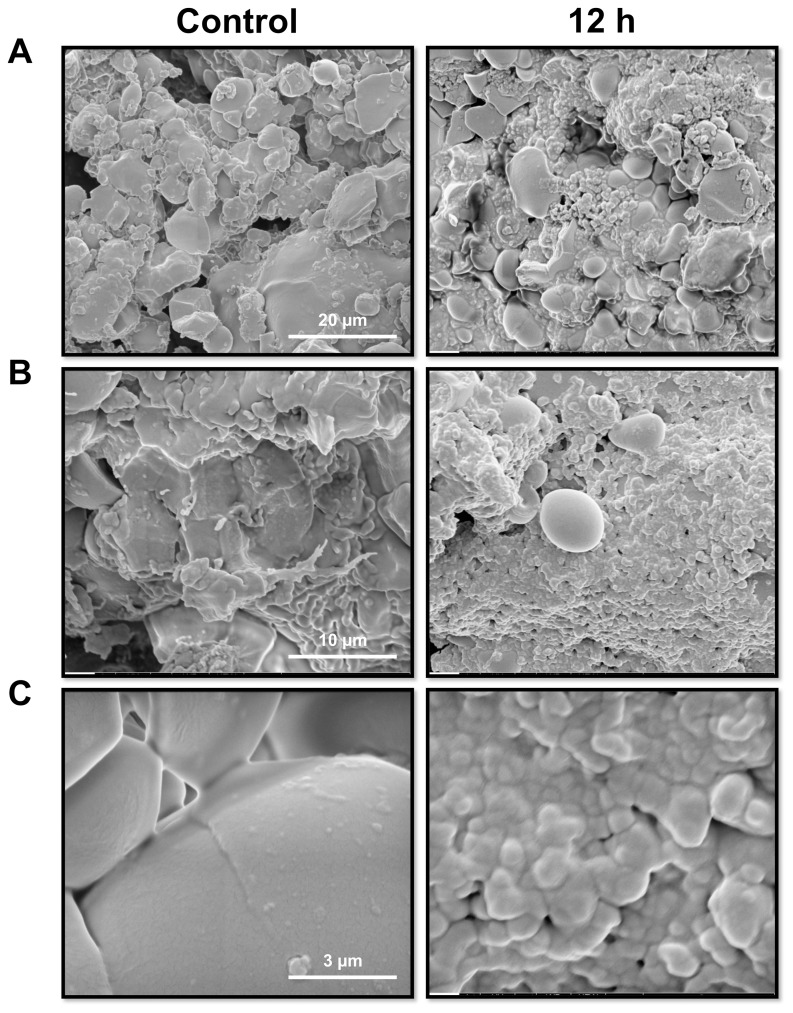
Scanning electron microscopy images showing the hydrolytic effect of GtCel12A on oat spelt. The left panels show images in different magnifications of the reaction control, and the right panels, the effects of the enzyme action after 12 hours at 50°C in pH 4.5. A- Scale bar of 20 µm. B- Scale bar of 10 µm. C- Scale bar of 3 µm.

The hydrolysis of oat spelt and filter paper (crystalline cellulose) by GtCel12A was examined by scanning electron microscopy. Following 12 hours of incubation, the control oat spelt (sample without enzyme treatment) displayed relatively uniformed size granules with smooth round surface. After hydrolysis by GtCel12A, the oat spelt showed a predominance of pitted surface with numerous small bulbous particles (Fig. 8). The differences in appearance exhibited by the control and enzyme-treated samples suggest that the surface of the large granules in oat spelt was digested by GtCel12A. This can be demonstrated in the set of images of figures 8A and 8B.

In figure 8C (higher magnification) one can observe in more detail the particle surface, revealing a smoother and more continuous surface in the control sample. In contrast, in the hydrolyzed sample, higher roughness and surface imperfections can be observed, with evidence of small granules resulting from the enzyme action.

**Figure 9:**
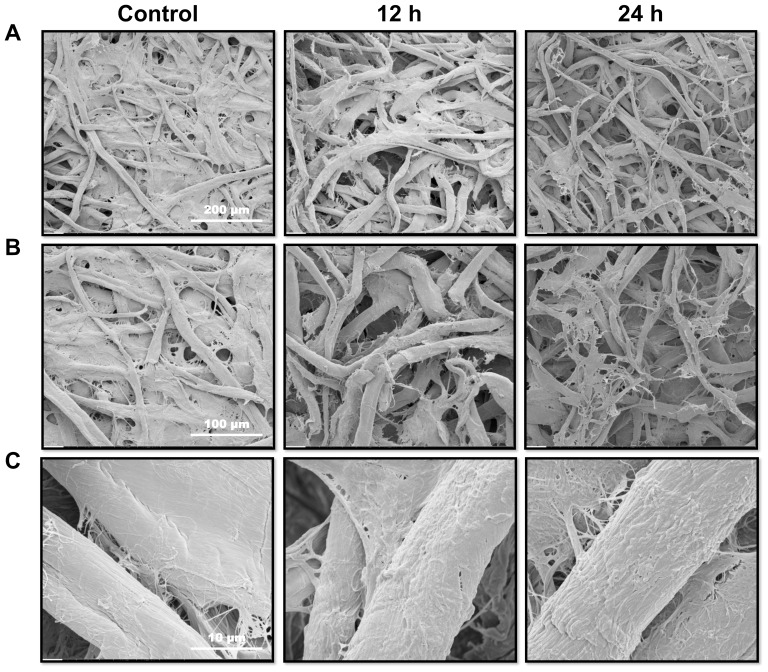
Scanning electron microscopy images showing the hydrolytic effect of GtCel12A on filter paper. Left panels, images in different magnifications of the reaction control; center panels, after 12 hours of enzyme action; right panels, after 24 hours of enzyme hydrolysis. A- Scale bar of 200 µm. B- Scale bar of 100 µm. C- Scale bar of 10 µm.

Filter paper was used as a model cellulosic substrate to demonstrate the effect of GtCel12A. At low magnification (Fig. 9A), the control sample displays a closed mesh of cellulose fibers. Following enzyme digestion, the mesh of fibers becomes more open, and the thinner fibers, which formed a continuous tissue filling the interstices between the thicker fibers, disappear, leaving behind looser fibers. Interstitial fibers that are not protected by the neighbors in the thick paper yarns are thus more susceptible to the enzyme action. Compared to 12 hours of hydrolysis, the fibers in the samples hydrolyzed for 24 hours are thinner and more shredded with a more opened mesh (Fig. 9B).

At high magnification (Fig. 9C), the fiber surface after hydrolysis is rougher and has more defects, when compared to the control sample. The exposure of smaller fibrils on the surface of the thick fibers following enzyme digestion can be attributed to the degradation of surrounding fibrils.

## Discussion

Previous studies showed that enzymes from GH12 family are active on a relatively wide range of substrates [18]–[21]. The GH12 endoglucanase from *Fomitopsis palustris* is most active on lichenan (β-1,3 and β-1,4 linkages), and is also active on barley β-glucan, pachyman (β-1,3), laminarin (β-1,3), CMC (β-1,4), pustulan (β-1,6), glucommanan from konjac and larch, and xyloglucan from tamarind (β-1,4) [22]. The *Phanerochaete chrysosporium* GH12 endoglucanase is active on CMC, amorphous cellulose, and minor activity was observed on xylan and mannan [23]. The GH12 enzyme from *Aspergillus japonicus* shows highest activity on β-glucan from barley and lichenan [20]. Previously Cel12A from *G. trabeum* was shown to be active on CMC with minor activities on birchwood xylan and PASC [10] and is also active on filter paper and Avicel (FGSC A1513) [24]. Using a broader range of substrates to determine substrate specificity, we showed that G. *trabeum* Cel12A has its highest activity on β-glucan under optimal conditions of pH 4.5 and 50°C. It also displays a high activity on lichenan (65% of the maximum activity), which agrees well with previous findings that shows a preference of GH12 family members for substrates with mixed linkages such as β-glucan and lichenan over polyssacharides with only β-1,4 linkages. The available results suggest that for GtCel12A and other members of GH12 family, the enzymatic activities are not limited to cellulose-based substrates [6], [22], [25]–[27]. Fungal enzymes from GH12 family do not possess CBM, and thus are thought to preferentially degrade amorphous cellulose rather than crystalline cellulose. It was recently shown that *Trichoderma harzianum* Cel12A incorporates important residues mediating interactions of the *Cellulomonas fimi* CBM with the amorphous regions of cellulose [28]. We speculate that similar molecular organization can facilitate interactions of *G. trabeum* Cel12A with none-crystalline polysaccharides. Furthermore, we and others [24] showed that GtCel12A displays activity, albeit low, towards the commercial cellulose Avicel. It should be noted that the commercial microcrystalline cellulose, Avicel (Fluka, St Louis, USA) and Sigmacell (Sigma-Aldrich, St Louis, USA) contains amorphous regions and are heterogeneous substrates. Previous studies reported crystallinity index of 92.97% for Avicel, while Sigmacell type 20 and type 101 show crystallinity of 91.54% and 54.70%, respectively, as determined by X-ray diffraction analysis [29], [30].

Temperature and pH profile for GtCel12A suggested that ithas an acidophilic character, since it retained about 40% of its activity even for the lowest pH tested (pH = 1) under the optimal temperature of 50°C.This feature might be of biotechnological interest, mainly for 2^nd^ generation ethanol production, which frequently involves processes run at acidic pHs.

Thermal stability assay reveals a very stable enzyme, with half-life of 84.6±3.5 hours at 50°C and retains about 30% of its activity even after 120 hours of incubation at 50°C and pH 4.5. These results differ from previous thermal stability results on testing of *G. trabeum* Cel12A against CMC in similar conditions, which showed a much shorter half-life of 2 hours [24].

Analysis by SEM showed that GtCel12A is active on filter paper, used as a model cellulosic substrate. The effect of enzyme digestion was evidenced by the degradation of the thinner and most vulnerable cellulose fibers and the emergence of defects on the fiber surface. Furthermore, analysis by SEM showed that GtCel12A is also active on oat spelt, a natural substrate rich in β-glucan. Mixed-linkage (1→3),(1→4)-β-D-glucan or β-glucans are important unbranched water-soluble polysaccharide present in plant cell walls [31]. β-glucans are unique to the order Poales [32], the taxonomic order that includes cereal grasses. β-glucans are tightly integrated with cellulose and other noncellulosic polysaccharides in the primary walls of growing cells [33]. This polysaccharide is mainly found in oat and barley but smaller amounts are present in other cereals such as corn, rice, rye, wheat and sorghum [34]–[36]. Thus, the ability to degrade β-glucans is biotechnologically important since biomass from grasses (miscanthus, switchgrass, corn stover and sugarcane) represent promising feedstocks for 2^nd^ generation biofuel production, especially in Brazil and the United States [37], [38].

## Conclusions

Detailed biochemical characterization showed that Cel12A of *G. trabeum* is highly active on β-glucan. The enzyme is thermal stable in acidic environment. Results from SEM analysis on the effects of GtCel12A on oat spelt are consistent with the assertion that GH12 enzymes prefer mixed linkage (1→3), (1→4)-β-D-glucan as substrate. Since β-glucans are often found in abundance in grasses, these results suggest that GH12 enzymes are potentially important in the conversion of grass-derived biomass into fermentable sugars.
